# Discordant sestamibi uptake in synchronous parathyroid carcinoma and adenoma

**DOI:** 10.1530/EDM-26-0001

**Published:** 2026-04-15

**Authors:** Qian Wang, Xiang Zhou, Ruigang Lu, Hong Shen, Bojun Wei

**Affiliations:** ^1^ Department of Thyroid and Neck Surgery, Beijing Chao-Yang Hospital, Capital Medical University, Beijing, China; ^2^ Department of Pathology, Beijing Chao-Yang Hospital, Capital Medical University, Beijing, China; ^3^ Department of Ultrasound, Beijing Chao-Yang Hospital, Capital Medical University, Beijing, China

**Keywords:** parathyroid carcinoma, ^99m^Tc-MIBI scintigraphy, internal jugular venous PTH sampling, synchronous tumors

## Abstract

**Summary:**

The simultaneous occurrence of parathyroid carcinoma, parathyroid adenoma, and papillary thyroid carcinoma is exceptionally rare. Herein, we present an uncommon case in which the right malignant parathyroid lesion was ^99m^Tc-MIBI-negative and the contralateral benign parathyroid lesion was ^99m^Tc-MIBI-positive, which was confirmed by pathological examination postoperatively. In addition, we performed bilateral internal jugular venous sampling of PTH (IJ PTH), and the results showed that the IJ PTH was higher on the side of ^99m^Tc-MIBI-negative lesion than on the other side. These findings suggest that ^99m^Tc-MIBI-negative lesions with ipsilateral high IJ PTH should be highly suspected of parathyroid carcinoma in the setting of patients with two suspicious parathyroid lesions detected by cervical ultrasonography or computed tomography. No germline mutations associated with hereditary endocrine tumors were identified by whole-exome sequencing.

**Learning points:**

- Parathyroid carcinoma can appear negative on ^99m^Tc-sestamibi scintigraphy; therefore, comprehensive evaluation with both ultrasound and computed tomography is essential.
- In cases with discordant imaging and multiple lesions, bilateral internal jugular venous parathyroid hormone sampling serves as a decisive adjunct to guide en bloc resection when carcinoma is suspected.
- Preoperative evaluation for concurrent thyroid nodules is recommended during parathyroid surgery planning to avoid the need for secondary neck procedures.

## Background

Parathyroid carcinoma (PC) is a rare endocrine malignancy, comprising less than 1% of primary hyperparathyroidism (PHPT) cases and posing a significant challenge for preoperative distinction from benign parathyroid adenoma (PA), as they often share similar clinical and biochemical presentations. Preoperative identification of suspected parathyroid carcinoma is paramount, as these cases should undergo en bloc resection to minimize risks of recurrence and metastasis (1). ^99m^Tc-sestamibi (MIBI) scintigraphy is a cornerstone for preoperative localization of hyperfunctioning parathyroid tissue. The coexistence of PC and PA, especially with synchronous thyroid carcinoma, is exceedingly uncommon, with only a handful of cases described (2, 3, 4, 5). This report presents a diagnostically intriguing case of a patient with severe PHPT due to synchronous, ipsilateral MIBI-negative PC and contralateral MIBI-positive PA, alongside an incidental papillary thyroid carcinoma (PTC), highlighting a discordant imaging pattern and the potential adjunctive role of bilateral internal jugular venous parathyroid hormone (PTH) sampling in such complex scenarios.

## Case presentation

A 55-year-old female presented with a two-year history of progressive low back pain. Laboratory evaluation revealed significant hypercalcemia (serum calcium: 3.01 mmol/L, reference range (RR): 2.11–2.52), elevated ionized calcium (1.67 mmol/L, RR: 1.09–1.30), a markedly high PTH level (321.5 pg/mL, RR: 18.5–88.0), vitamin D deficiency (25-hydroxyvitamin D: 11.77 ng/mL, RR: 30–100), and increased alkaline phosphatase (167 U/L, RR: 35–100). Other biochemical indices were within normal limits.

## Investigation

**Figure 1:**
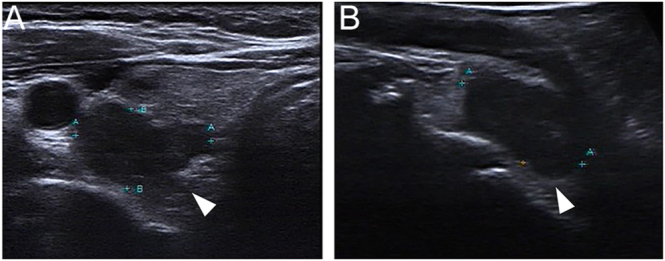
A 55-year-old female had a history of low back pain for two years. Preoperative examination showed abnormal serum calcium (3.01 mmol/L, reference range (RR): 2.11–2.52 mmol/L), ionized calcium (1.67 mmol/L, RR: 1.09–1.30 mmol/L), parathyroid hormone (PTH) (321.5 pg/mL, RR: 18.5–88.0 pg/mL), 25-hydroxyvitamin D (11.77 ng/mL, RR: 30–100 ng/mL), and alkaline phosphatase (ALP) (167 U/L, RRreference range: 35–100 U/L) levels, in conjunction with normal levels of other indicators. Dual-phase ^99m^Tc-sestamibi planar imaging at 10 min (A) and 2 h (B) after intravenous injection of ^99m^Tc-MIBI (15 mCi) showed persistent focal uptake of the tracer in a lesion below the left thyroid lobe (B, black arrowhead).

**Figure 2:**
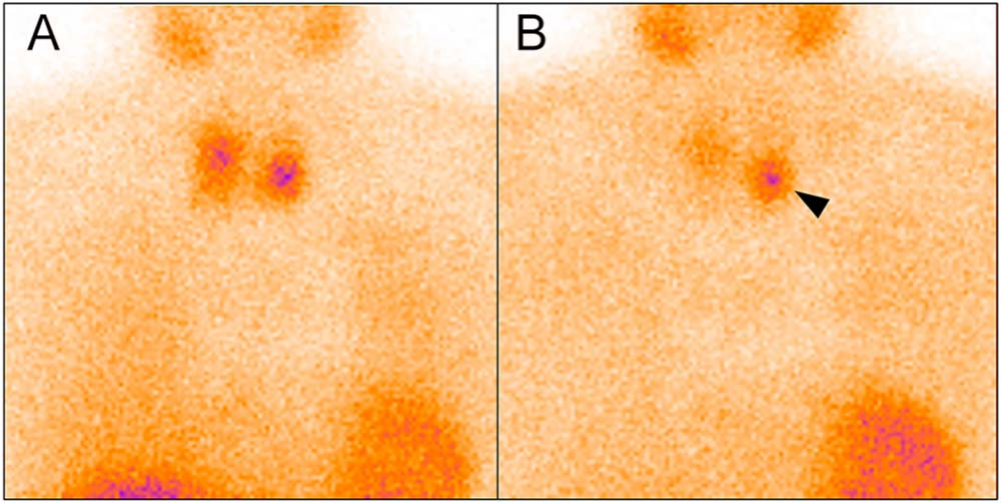
Dual-phase ^99m^Tc-sestamibi scintigraphy with single-photon emission computed tomography/computed tomography (SPECT/CT) imaging showed high uptake of ^99m^Tc-MIBI in the area of the left inferior pole (A, red arrowhead), without abnormal uptake of the tracer in the contralateral neck section (A, red arrow). Dual-energy enhanced CT showed a right 2.0 × 1.1 cm suspected parathyroid lesion (B, red arrow) and a left 1.4 × 1.2 cm suspected parathyroid lesion (B, red arrowhead).

**Figure 3:**
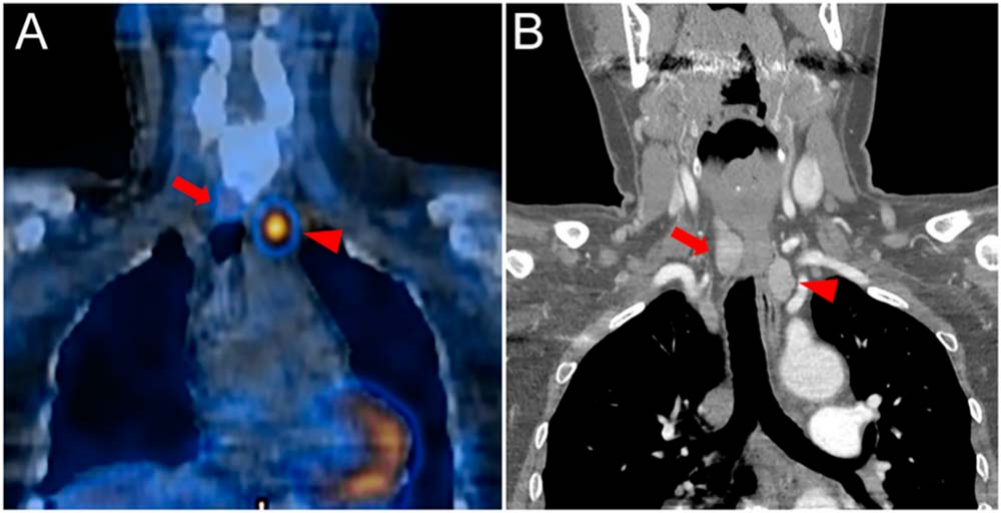
Cervical ultrasonography demonstrated a hypoechoic lesion in the middle dorsal of the right thyroid lobe (A, white arrowhead), measuring 2.5 × 1.6 cm, which was suspicious for parathyroid carcinoma (PC) because of its irregular morphology. There was another hypoechoic image below the left thyroid lobe (B, white arrowhead). It measured 2.0 × 1.3 cm, with a clear border and regular shape, which was considered a parathyroid adenoma (PA).

Dual-phase ^99m^Tc-MIBI planar imaging (6) showed persistent focal tracer uptake inferior to the left thyroid lobe at both 10-min and 2-h delays (Fig. 1A, 1B). Subsequent SPECT/CT fusion imaging confirmed this uptake corresponded to the left inferior lesion but revealed no abnormal tracer concentration on the right side (Fig. 2A). A dual-energy enhanced neck CT identified two suspicious lesions: located posterior to the right thyroid lobe (2.0 × 1.1 cm) and inferior to the left thyroid lobe (1.4 × 1.2 cm) (Fig. 2B). Cervical ultrasonography characterized the right lesion as a 2.5 × 1.6 cm hypoechoic mass with irregular borders, suspicious for PC, while the left lesion was a well-defined, 2.0 × 1.3 cm hypoechoic nodule, suggestive of a typical PA (Fig. 3A, 3B).

Given the discordance between functional (MIBI-negative right lesion) and anatomical imaging (clearly visualized bilateral lesions), and the high suspicion for malignancy on the right, bilateral internal jugular (IJ) venous PTH sampling was performed intraoperatively (7). Baseline IJ PTH levels were significantly asymmetric: 1,027.5 pg/mL on the right side vs 322.9 pg/mL on the left.

## Treatment

The combination of a right-sided, irregularly bordered mass on neck ultrasound (highly suggestive of carcinoma) and significantly higher parathyroid hormone levels in the right internal jugular vein than the left provided compelling evidence for parathyroid carcinoma. The patient underwent en bloc resection (1) of the right parathyroid mass with ipsilateral thyroid lobectomy and left parathyroidectomy. Intraoperatively, the left upper and right lower parathyroid glands were identified and appeared normal. Ten minutes after resection of all suspected lesions, IJ PTH levels dropped appropriately to 78.8 pg/mL (right) and 65.4 pg/mL (left).

## Outcome and follow-up

**Figure 4:**
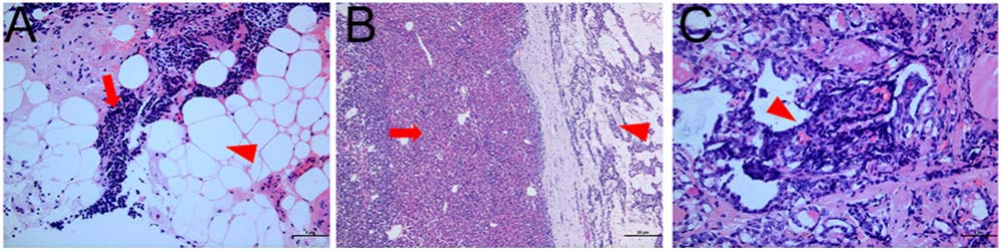
The patient underwent en bloc resection of the right parathyroid mass, ipsilateral thyroid lobectomy, and left parathyroidectomy. The left upper and right lower parathyroid glands were identified normally. Bilateral IJ PTH was measured at baseline and 10 min after removal of all lesions. The right IJ PTH dropped to 78.8 pg/mL from 1027.5 pg/mL and the left IJ PTH dropped to 65.4 pg/mL from 322.9 pg/mL. The histopathologic section revealed the right lesion as a PC with evidence of tumor cell (A, red arrow) invasion into adjacent fatty tissue (A, red arrowhead) (hematoxylin and eosin stain (H&E), ×200). The left parathyroid gland was a PA with evidence of normal parathyroid tissue (B, red arrowhead), seen next to the parathyroid tumor (B, red arrow) (H&E, ×50). (C) An incidental 0.2 cm papillary thyroid carcinoma was revealed in the right thyroid lobe (C, red arrowhead) (H&E, ×200).

Histopathological examination confirmed the diagnosis. The right-sided lesion was a PC, showing evidence of tumor cell invasion into the surrounding adipose tissue (Fig. 4A). The left-sided lesion was a PA, with adjacent normal parathyroid tissue identified (Fig. 4B). Furthermore, pathological examination of the right thyroid lobe revealed an incidental 0.2 cm focus of papillary thyroid carcinoma (Fig. 4C). Whole-exome sequencing was performed to exclude multiple endocrine neoplasia syndromes, and no germline mutations associated with hereditary endocrine tumors were identified.

At the one-year postoperative follow-up, the patient was normocalcemic (serum calcium: 2.45 mmol/L, ionized calcium: 1.25 mmol/L) with a normal PTH level (80.1 pg/mL) and alkaline phosphatase (81 U/L).

## Discussion

This case presents three remarkable and intersecting points of clinical interest. First, it reports the rare synchronous occurrence of PC, PA, and PTC. To date, only seven cases of concurrent PC and PA have been documented, and merely two included concomitant thyroid cancer (8, 9). Our case adds to this limited literature. Second, and most notably, it demonstrates a discordant ^99m^Tc-MIBI uptake pattern: the malignant parathyroid lesion was MIBI-negative, while the benign adenoma showed classic persistent uptake. This phenomenon, though uncommon, has been sporadically reported in PCs and is attributed to reduced mitochondria-rich oxyphil cells, leading to rapid tracer washout (10). This case serves as a critical reminder that a ‘negative’ MIBI scan does not rule out a hyperfunctioning, and possibly malignant, parathyroid lesion, especially when anatomical imaging suggests otherwise. Third, we utilized bilateral internal jugular venous PTH sampling, which provided compelling functional lateralization. The significantly higher IJ PTH on the side of the MIBI-negative lesion strongly indicated it as the dominant source of PTH hypersecretion, aligning with the final pathological diagnosis of carcinoma. This technique, as supported by prior studies (7), can be a valuable intraoperative adjunct in complex cases to confirm the laterality of hypersecretion. The presence of an incidental PTC, while likely coincidental, highlights the importance of preoperative evaluation for concurrent thyroid nodules to reduce the risk of complications from a second neck exploration.

## Declaration of interest

The authors declare that there is no conflict of interest that could be perceived as prejudicing the impartiality of the work reported.

## Funding

This work was supported by the Beijing Medical Award Foundation (XYJL-2022-0028-0003).

## Patient consent

Written informed consent for publication of their clinical details and clinical images was obtained from the patient.

## Author contribution statement

QW wrote the manuscript and managed the patient’s clinical evaluation, treatment, and follow-up. RL performed the ultrasound and imaging evaluations. HS and BW performed the surgery. XZ established the pathological diagnosis. All authors reviewed and approved the final manuscript.
